# Short-day photoperiod enhances graft success by increasing auxin accumulation

**DOI:** 10.1080/15592324.2026.2648973

**Published:** 2026-03-31

**Authors:** Kihwan Kim, Jongbae Son, Won-Chan Kim

**Affiliations:** a Department of Applied Biosciences, Kyungpook National University, Daegu, Republic of Korea; b Department of Integrative Biology, Kyungpook National University, Daegu, Republic of Korea

**Keywords:** *Arabidopsis thaliana*, auxin biosynthesis, photoperiod, plant grafting, polar auxin transport

## Abstract

Plant grafting is a key horticultural practice used to promote agricultural performance by improving crop productivity and enhancing resistance to biotic and abiotic stresses. Successful graft union formation depends on tissue adhesion, callus formation, and vascular reconnection, which are regulated primarily by auxin signaling. Although environmental factors influence grafting union formation by modulating hormonal signaling, the role of photoperiod in graft union formation remains poorly understood. Here, we demonstrate that short-day (SD) photoperiods markedly enhance grafting success in *Arabidopsis thaliana* by promoting hypocotyl elongation and increasing auxin accumulation at the graft junction. DR5:GUS reporter assays revealed strong photoperiod-dependent differences in auxin responsiveness, with the highest auxin levels under SD conditions and substantially reduced levels under long-day (LD) and medium-day (MD) conditions. Exogenous indole-3-acetic acid (IAA) treatment restored the grafting success rate under LD and MD conditions, whereas inhibition of polar auxin transport by 2,3,5-triiodobenzoic acid (TIBA) significantly reduced grafting success under SD conditions. Notably, TIBA treatment did not lead to a further significant reduction under LD and MD conditions, where endogenous auxin levels were already limiting, confirming the necessity of localized auxin accumulation at the graft junction. Furthermore, transferring grafted seedlings from LD to SD conditions restored grafting success, highlighting the plasticity of light-responsive transcriptional pathways that regulate auxin biosynthesis. Together, these findings reveal that photoperiod enhances grafting success primarily by increasing endogenous auxin accumulation, which in turn enables efficient auxin transport and the activation of auxin-mediated regenerative programs at the graft junction. This work establishes photoperiod as a key environmental determinant of grafting efficiency and provides a framework for optimizing grafting outcomes through the modulation of light conditions and hormonal signaling.

## Introduction

Plant grafting, which is defined as the union of a scion and rootstock, is one of the most widely used horticultural practices and is essential in modern agriculture to improve crop productivity, yield, stress tolerance, and disease resistance.^1-3^ Over the past century, plant grafting has expanded from its traditional application in woody perennials to a wide range of herbaceous crops, highlighting its increasing importance in modern agricultural production.^4^^,^^5^ With the increasing adoption of grafting in modern agriculture, diverse strategies have been developed to enhance grafting success, including methodological innovations, the exogenous application of phytohormones, and the regulation of environmental factors. Advances such as micrografting techniques and automated grafting systems have increased precision and reproducibility.^6-8^ Exogenous application of phytohormones has been employed to stimulate cell division, callus formation, and vascular differentiation at the graft junction.^9-11^ Among these approaches, the regulation of environmental factors, such as humidity,^12^^,^^13^ temperature,^14^^,^^15^ and light quality and quantity,^16^^,^^17^ has consistently emerged as a decisive factor influencing grafting success. These environmental factors modulate endogenous hormone signaling and transcriptional responses, orchestrating the sequential processes of graft union formation.^18-20^ Although these strategies contribute to enhancing grafting success, understanding how environmental factors interact with endogenous hormonal signals remains essential for elucidating the molecular mechanisms of graft union formation.

Recent studies have demonstrated that auxin acts as a key endogenous regulator in graft union formation.^7^^,^^18^^,^^20^^,^^21^ After grafting, the establishment of polar auxin transport mediates the directional flow of auxin from the scion to the rootstock, leading to localized auxin accumulation at the graft junction.^15^^,^^22^ The auxin gradient facilitates graft union formation by orchestrating sequential cellular processes under the regulation of gene‒hormone signaling networks.^23^^,^^24^ At the molecular level, graft union formation is orchestrated through sequential phases, including tissue adhesion, callus formation, and vascular reconnection, under the control of hormone signaling networks. At the initial stage of tissue adhesion, wound-induced AP2/ERF transcription factors, such as WIND1, promote cellular dedifferentiation and induce the expression of WOX13.^25^^,^^26^ WOX13 regulates the transcription of cell wall-modifying enzymes, including endo-β-1,4-glucanase (GH9B3, also known as CEL3) and expansins (EXP), facilitating cell wall remodeling and promoting tissue adhesion.^27^ Moreover, WIND1 enhances cytokinin signaling, which reactivates cell division and reinforces tissue adhesion between the scion and rootstock, providing the foundation for successful graft union formation.^28^ At the stage of callus formation, auxin promotes the expression of ERF115, activating cell cycle regulators and resuming cell division.^29^^,^^30^ Simultaneously, auxin-induced DOF transcription factors, such as HCA2 and TMO6, activate cell division and contribute to regenerative growth.^31^^,^^32^ Moreover, auxin accumulation in scions induces ANAC071, further increasing cell division at the graft junction, whereas the disruption of auxin influx from the rootstock upon cutting induces the activation of RAP2.6L, which modulates cell cycle progression and coordinates cell division. ^24^ These auxin-regulated networks orchestrate sustained callus formation, establishing the cellular framework necessary for vascular reconnection. At the stage of vascular reconnection, auxin enhances cambial proliferation by inducing the expression of WOX4, a key downstream regulator of cambial proliferation.^33^ WOX4 is a known downstream target of the PXY signaling pathway and acts as a central mediator of vascular development.^34^^,^^35^ Moreover, auxin coordinates the spatial patterning of xylem‒phloem differentiation through interactions with cytokinin and gibberellin. Cytokinin stabilizes vascular patterns by regulating auxin polar transport.^36^ The auxin and cytokinin interact through a mutually inhibitory mechanism, with auxin predominantly promoting xylem remodeling, whereas cytokinin promotes phloem remodeling.^37^^,^^38^ The cross-regulation of auxin and cytokinin establishes a complementary developmental process that ensures the coordinated production of xylem and phloem during vascular reconnection. Gibberellin acts with auxin to determine the fate of cambial stem cell daughters. Gibberellin enhances PIN1-dependent polar auxin transport, which broadens the auxin maximum from the xylem side of the cambium towards the phloem.^39^^,^^40^ The redistribution of auxin promotes xylem differentiation in the xylem-side stem cell daughter, regulating the ratio of xylem and phloem production. Together, graft union formation is orchestrated by auxin signaling, which sequentially regulates tissue adhesion, callus formation, and vascular reconnection.

Auxin biosynthesis and signaling are modulated by environmental signals through the PhyB–PIF4 module.^41-43^ PhyB acts as a molecular hub that perceives both red/far-red light and ambient temperature, integrating environmental signals into the regulation of auxin biosynthesis and signaling.^20^^,^^44^^,^^45^ Exposure to elevated ambient temperature promotes the thermal inactivation of PhyB, alleviating PhyB-mediated repression of PIF4, leading to PIF4 stabilization and nuclear accumulation.^46^^,^^47^ The PIF4 subsequently transcriptionally upregulates the YUCCA8 gene, which catalyzes the rate-limiting step in auxin biosynthesis, resulting in increased endogenous auxin levels.^48^^,^^49^ These regulatory mechanisms supported previous studies that elevated ambient temperature contributes to enhanced grafting success rate by promoting auxin biosynthesis and signaling.^15^^,^^50^ Similarly, photoperiod alteration regulates PhyB activity, which modulates the stability and nuclear accumulation of PIF4 through degradation and sequestration, influencing auxin accumulation and signaling.^51^^,^^52^ Under Short-day (SD) conditions, suppression of PhyB photoactivation leads to the stabilization and nuclear accumulation of PIF4, activating downstream auxin signaling. In contrast, under long-day (LD) conditions, photoactivated PhyB promotes PIF4 degradation, limiting its transcriptional activity. This regulatory mechanism is consistent with previous studies indicating that the PIF4 circadian gene expression cycle is dependent on PhyB activity, suggesting that photoperiod-dependent regulation of the PhyB–PIF4 module dynamically regulates endogenous auxin biosynthesis and signaling.^53-55^ However, the role of the photoperiod in regulating auxin dynamics during graft union formation remains poorly understood. Therefore, this study aims to elucidate how photoperiod orchestrates the molecular mechanisms underlying auxin dynamics in graft union formation.

## Materials and methods

### Plant materials and growth conditions

*Arabidopsis thaliana* (ecotype Columbia-0; Col-0) was used as the wild type (WT). The plants were grown in a growth chamber at 23 °C under long-day (LD; 16 h light/8 h dark), medium-day (MD; 12 h light/12 h dark), or short-day (SD; 8 h light/16 h dark) conditions with a light intensity of approximately 100 μmol m^−2^ s^−1^. Seeds were surface sterilized with a solution containing 4% (v/v) NaOCl and 0.05% (v/v) Triton X-100 for 20 min, then rinsed three times with sterile distilled water, followed by treatment with 70% (v/v) ethanol for 1 min, and finally rinsed ten times with sterile distilled water. The sterilized seeds were subjected to a 2-d cold treatment in the dark at 4 °C before sowing. After cold treatment, the seeds were germinated on half-strength Murashige and Skoog (^1^/_2_ × MS) medium supplemented with 0.05% (w/v) MES and 0.8% (w/v) sucrose, adjusted to pH 5.8 with a standardized 2 N NaOH solution.

### Construction of DR5:GUS binary vector and *Arabidopsis* transformation

The DR5:GUS binary vector was constructed to monitor auxin accumulation and signaling.^56-58^ To construct the DR5:GUS binary vector, DNA oligonucleotides corresponding to the DR5 promoter were synthesized by Integrated DNA Technology (IDT). The synthesized DR5 promoter fragments were inserted into pCB308^59^using the restriction enzymes *Xba*I and *Spe*I. The constructed DR5:GUS binary vector was introduced into *A. thaliana* via *Agrobacterium tumefaciens* GV3101-mediated floral dip transformation.^60^ For preliminary screening of transformants, seeds of T_0_ transgenic plants were grown on ^1^/_2_ MS medium supplemented with glufosinate (10 μg/mL) based on the glufosinate resistance gene present in pTK-BMLC. After growth at 23 °C for 5 d under LD conditions, glufosinate-resistant individuals exhibiting green cotyledons were selected as T_1_ transgenic plant candidates.

### Genomic DNA extraction and validation of transgenic plants

For validation of the insertion of the DR5:GUS expression cassette in the transgenic plants, genomic DNA was extracted from rosette leaves of T_1_ transgenic plant candidates and amplified via polymerase chain reaction (PCR) using specific primers (Supplemental Table 1). The genomic DNA (gDNA) was extracted according to our previous study.^61^ Briefly, the fresh rosette leaves from T_1_ transgenic plant candidates were ground using a plastic pestle and added to 350 μL of modified Edwards solution (400 mM LiCl, 200 mM Tris–HCl, 25 mM EDTA, and 1% (v/v) SDS, pH 9.0). After centrifugation at 13,000 rpm for 10 min at 25 °C, 200 μL of the supernatant was transferred to a new Eppendorf tube (E-tube) and mixed with an equal volume of isopropanol. The mixture was then centrifuged at 13,000 rpm for 20 min at 25 °C. The supernatant was discarded, and 500 μL of 70% (v/v) ethanol was added to resuspend the pellet, followed by centrifugation at 13,000 rpm for 5 min at 25 °C. The pellet was dissolved in 50 μL of distilled water.

The PCR conditions were as follows: initial denaturation at 95 °C for 5 min; 34 cycles of denaturation at 95 °C for 30 sec, annealing at 55 °C for 30 sec, and elongation at 72 °C for 1 min; then an extension step at 72 °C for 5 min. The resulting PCR products were visualized using DNA electrophoresis on a 0.8% (w/v) agarose gel.

### Evaluation of grafting success rate

*A. thaliana* seeds were grown vertically on ^1^/_2_ MS medium in a growth chamber for 5 d under three different photoperiod conditions. For each photoperiod condition, 5-d-old *A. thaliana* scions and rootstocks with elongated hypocotyls of similar size were grafted. The upper portion of the hypocotyls was cut with a sterile razor blade.^62^^,^^63^ For each photoperiod condition, a total of 165 WT/WT grafts were used. These grafts were divided into three treatment groups (DMSO, IAA, and TIBA), with 55 grafts assigned to each treatment. The grafted seedlings were transferred to fresh ^1^/_2_ MS medium supplemented with 14 mM DMSO, 0.001 μM IAA, or 10 μM TIBA (IAA and TIBA were dissolved in DMSO and added to the ^1^/_2_ MS medium; therefore, an equivalent volume of DMSO was used as a solvent control.) according to the photoperiod in which the seedlings were grown for 5 d. To assess the effects of photoperiod changes and postgraft IAA application on grafting success, 44 WT/WT grafts were grown for 5 d under LD conditions and transferred to fresh ^1^/_2_ MS medium supplemented with 14 mM DMSO, 0.001 μM IAA under different photoperiod conditions. Graft union formation was examined 10 d postgrafting using an Olympus BX52F2 microscope.

### Determination of hypocotyl length

*A. thaliana* seedlings were grown for 5 d or 10 d postgrafting under different photoperiod conditions, treated with DMSO, IAA, and TIBA, and photographed using a Leica EZ4E microscope. The hypocotyl length was measured using Image J software as the distance from the collet of the root hairs to the ‘v’ made by the cotyledon shoulder.

### Histochemical GUS staining

T_1_ DR5:GUS transgenic plants self-pollinated to produce T_2_ progeny seeds. T_2_ seedlings were grown vertically for 5 d under different photoperiod conditions and treated with DMSO, IAA, or TIBA. The histochemical GUS staining was conducted according to previous studies.^64^^,^^65^ Briefly, seedlings were subsequently incubated overnight at 37 °C in GUS staining solution [50 mM NaPO_4_, 10 mM EDTA, 2  mM K_4_Fe(CN)_6_, 2 mM K_3_Fe(CN)_6_, 0.1% (v/v) Triton X-100, and 2.5 g/L X-gluc (5-bromo-4-chloro-3-indoly-β-D-glucuronic acid), pH 7.0]. Chlorophyll and other pigments were removed by sequential incubation in increasing ethanol concentrations (25%, 50%, 75%, and 100%) at 37 °C, with each concentration applied overnight. The chlorophyll-free seedlings were photographed using a Leica EZ4E microscope.

### Quantification of GUS activity

T_2_ DR5:GUS transgenic plants were grown for 5 d under different photoperiod conditions and treated with DMSO, IAA, or TIBA. Three DR5:GUS transgenic plants were used per sample. The samples were frozen in liquid nitrogen, and total protein was extracted using 100 μL of extraction buffer [100 mM KPO_4_, 0.1 mM EDTA, 10% (v/v) glycerol, and 1% (v/v) Triton X-100]. After centrifugation at 13,000 rpm for 10  min at 4 °C, the supernatant was transferred to a new E-tube, and the protein concentration was determined using the Bradford assay.^66^ GUS activity was measured using the Synergy HTX microplate spectrofluorometer for 4-methylumbelliferone, a degradation product of 4-methylumbelliferyl β-D-glucuronide (MUG). GUS activity was expressed as μmol of 4-methylumbelliferone per min per milligram of protein.

### Statistical analysis

The data were analyzed by one-way analysis of variance (ANOVA) using SAS version 9.4. Multiple comparisons were conducted using Tukey’s honestly significant difference (HSD) test at a significance level of *p* < 0.05. The data are presented as the mean ± standard deviation (SD).

## Results

### Photoperiod-dependent conditions for grafting success and scion growth of *A. thaliana* seedlings

**Figure 1.**
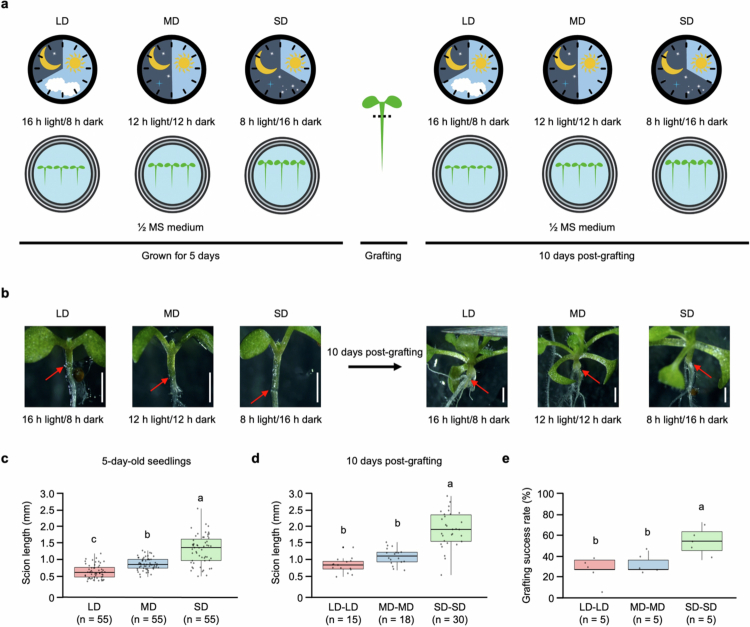
Effects of photoperiod on the grafting success rate and scion growth in seedlings of *Arabidopsis thaliana*. (a) Schematic overview of the experimental design. Seedlings were initially grown for 5 d under long-day (LD; 16 h light/8 h dark), medium-day (MD; 12 h light/12 h dark), or short-day (SD; 8 h light/16 h dark) conditions before grafting. After grafting, the seedlings were grown under the same respective photoperiod conditions for an additional 10 d. (b) Representative images of graft unions immediately after grafting (left panels) and 10 d postgrafting (right panels) under each photoperiod condition. The red arrows indicate the graft union site. Scale bars = 1 mm. (c) Scion length of 5-d-old seedlings before grafting under each photoperiod condition. Each biological replicate represents an individual seedling. (d) Scion length of 10 d postgrafting. Each biological replicate represented an individual grafted seedling that resulted in successful graft union formation. (e) Quantification of the grafting success rate under each photoperiod condition. Each biological replicate (*n* = 5 per group) represented the mean grafting success rate calculated from 11 individual grafted seedlings that resulted in successful graft union formation (total of 55 grafts per condition). The data are presented as mean ± standard deviation (SD). Different letters indicate statistically significant differences among the data as determined by one-way ANOVA (*p* < 0.05).

To investigate whether photoperiod conditions play a critical role in determining grafting success, *A. thaliana* seedlings were grown for 5 d under long-day (LD; 16 h light/8 h dark), medium-day (MD; 12 h light/12 h dark), or short-day (SD; 8 h light/16 h dark) conditions before grafting, after which the grafted seedlings were maintained under the same respective photoperiod conditions for an additional 10 d (Figure 1a). The formation of the graft union was verified through representative images of the graft junction photographed immediately after grafting and 10 d postgrafting, and grafting success was evaluated based on these observations (Figure 1b). Seedlings grown for 5 d under different photoperiod conditions exhibited significant differences in hypocotyl length, with the longest hypocotyl length observed under SD conditions, followed by MD and LD conditions (Figure 1c). At 10 d after grafting, the hypocotyl length remained significantly longest in SD condition, whereas no significant difference was detected between the LD and MD conditions (Figure 1d). These findings were consistent with the grafting success rate, which was highest under SD conditions, whereas no significant difference was observed between LD and MD conditions (Figure 1e).

### Photoperiod-dependent modulation of auxin response in *A. thaliana* seedlings

Photoperiod-dependent modulation of endogenous auxin accumulation, together with the regulation of auxin signaling through polar auxin transport, was hypothesized to play a critical role in controlling hypocotyl elongation. To test this hypothesis, the interaction between exogenous IAA and TIBA treatment was investigated under different photoperiod conditions. First, to determine the optimal treatment range of IAA or TIBA, *A. thaliana* seedlings were treated with exogenous IAA (0.001–0.1 μM) or TIBA (0.1–10 μM) under different photoperiod conditions for 5 d (Supplemental Figure 1). Treatment with 0.1 μM IAA resulted in greater inhibition of hypocotyl length compared to lower IAA concentrations (Supplemental Figure 1a and b), which is consistent with previous studies indicating that high concentrations of exogenous auxin suppress seedling growth.^67^^,^^68^ In contrast, treatment with 0.1 μM TIBA had no significant effect on hypocotyl length compared to the DMSO as a control, whereas higher TIBA concentrations induced a gradual inhibition in hypocotyl length (Supplemental Figure 1a). This pattern is consistent with previous studies,^69-71^ which demonstrated that the inhibition of polar auxin transport leads to characteristic morphological alterations, including impaired root gravitropism and reduced root growth (Supplemental Figure 1b), confirming the effective activity of TIBA. Based on these observations, an IAA concentration of 0.001–0.01 μM and a TIBA concentration of 1–10 μM were selected for subsequent analyses to investigate photoperiod-dependent auxin responsiveness while minimizing excessive growth inhibition caused by exogenous treatment.

**Figure 2.**
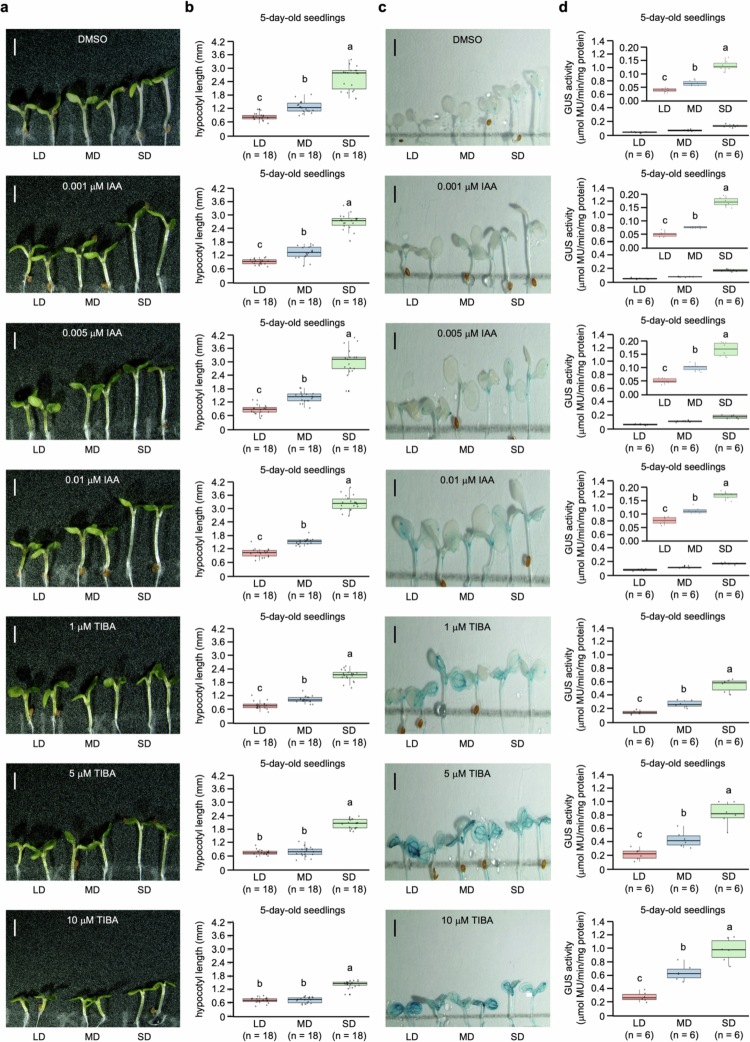
Photoperiod-dependent regulation of auxin-induced hypocotyl length in seedlings of *Arabidopsis thaliana*. (a) Representative images of 5-day-old seedlings grown under different photoperiod conditions and treated with different concentrations of IAA (0.001, 0.005, or 0.01 μM), TIBA (1, 5, or 10 μM), or DMSO (14 mM) as a control. Scale bars = 1 mm. (b) Quantification of hypocotyl length in seedlings grown under different photoperiod conditions. The data are presented as mean ± standard deviation (SD). Each biological replicate (*n* = 18 per group) represents an individual seedling. Different letters indicate statistically significant differences among the data as determined by one-way ANOVA (*p* < 0.05). (c) Representative histochemical GUS staining images of DR5:GUS transgenic seedlings grown under different photoperiod conditions and treated with different concentrations of IAA or TIBA. Scale bars = 1 mm. (d) Quantification of GUS enzymatic activity in DR5:GUS transgenic seedlings grown under different photoperiod conditions. Each biological replicate (*n* = 6 per group) represented the mean calculated from 3 individual DR5:GUS transgenic seedlings. The data are presented as mean ± standard deviation (SD). Different letters indicate statistically significant differences among the data as determined by one-way ANOVA (*p* < 0.05).

The photoperiod-mediated auxin effect on hypocotyl elongation was investigated by measuring hypocotyl length and auxin-responsive reporter (DR5) activity in DR5:GUS transgenic seedlings treated with IAA or TIBA under different photoperiod conditions (Figure 2). A distinct photoperiod-dependent pattern of hypocotyl length was observed under DMSO, IAA, or TIBA (Figure 2a, b). The hypocotyl length was longest in the seedlings grown under SD conditions, intermediate under MD conditions, and shortest under LD conditions, regardless of IAA or TIBA treatment. Consistent with these morphological patterns, DR5:GUS transgenic seedlings exhibited photoperiod-dependent differences in auxin responsiveness to both IAA and TIBA treatment (Figure 2c, d), with the highest GUS activity under SD conditions, intermediate GUS activity under MD conditions, and the lowest GUS activity under LD conditions. This GUS activity pattern is consistent with previous studies demonstrating that reduced light promotes auxin biosynthesis.^72-74^ Interestingly, despite significant differences in auxin responsiveness, no significant difference in hypocotyl length was observed in the TIBA treatment (5 and 10 μM) under LD and MD conditions (Figure 2b, d). These results suggest that downstream processes, such as polar auxin transport, may act as a bottleneck limiting the translation of auxin signaling into growth, which is consistent with previous studies,^75^^,^^76^ indicating that auxin elevates PIN abundance at the plasma membrane by inhibiting the internalization step of constitutive PIN cycling while promoting auxin efflux through a vesicle trafficking-dependent pathway. Moreover, the strong inhibition of polar auxin transport by high concentrations of TIBA, which enhances plasma membrane internalization and consequently increases GUS activity.^77^^,^^78^ These findings support the mechanisms by which hypocotyl growth is regulated through elevated auxin levels and spatial distribution mediated by polar auxin transport.

**Figure 3.**
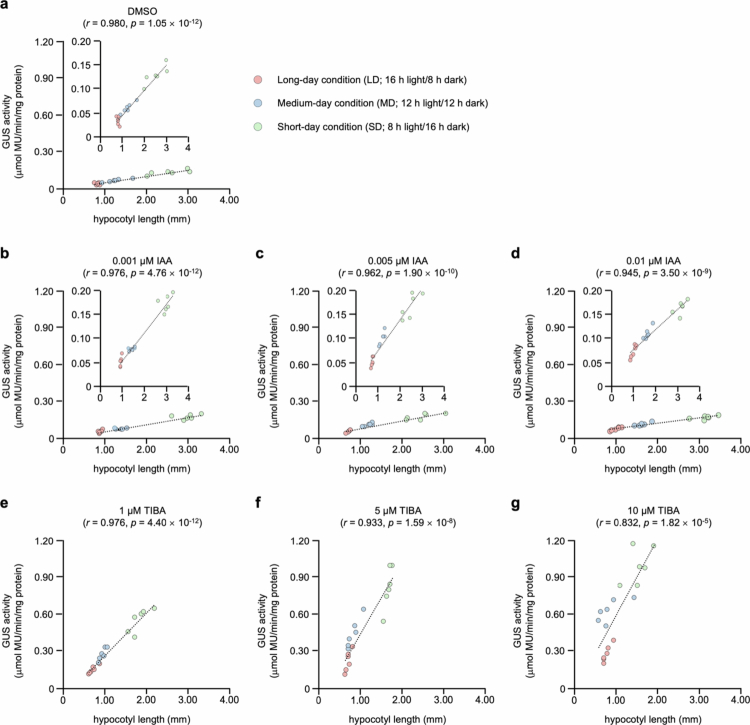
Scatterplots and correlations between hypocotyl length and GUS activity under different photoperiod conditions and treated with IAA or TIBA. Scatter plots exhibited the association between hypocotyl length and GUS activity in DR5:GUS transgenic seedlings grown under different photoperiod conditions. Each symbol represents the mean hypocotyl length from three individual DR5:GUS transgenic seedlings and the corresponding GUS activity (*n* = 6). The solid lines indicate the best-fit linear regression. Pearson correlation coefficients (*r*) and *p*-values were calculated to evaluate the relationship between hypocotyl length and GUS activity.

**Figure 4.**
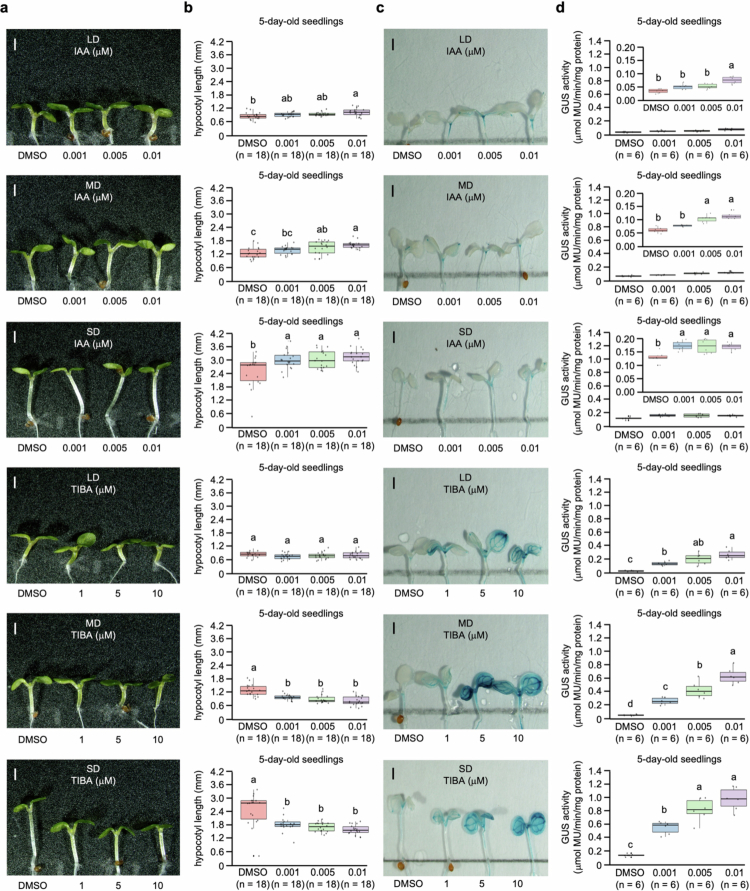
Differential responses to auxin and auxin transport inhibitors under different photoperiods in seedlings of *Arabidopsis thaliana*. (a) Representative images of 5-day-old seedlings grown under different photoperiod conditions on ^1^/_2_ MS medium containing increasing concentrations of IAA (0.001–0.01 μM) or TIBA (1–10 μM). Scale bars = 1 mm. (b) Quantification of hypocotyl length in seedlings grown with different concentrations of IAA or TIBA. The data are presented as mean ± standard deviation (SD). Each biological replicate (*n* = 18 per group) represents an individual seedling. Different letters indicate statistically significant differences among the data as determined by one-way ANOVA (*p* < 0.05). (c) Representative histochemical GUS staining images of DR5:GUS transgenic seedlings grown under different photoperiod conditions on ^1^/_2_ MS medium containing increasing concentrations of IAA (0.001–0.01 μM) or TIBA (1–10 μM). Scale bars = 1 mm. (d) Quantification of GUS enzymatic activity in DR5:GUS transgenic seedlings grown under different photoperiod conditions on ^1^/_2_ MS medium containing increasing concentrations of IAA (0.001–0.01 μM) or TIBA (1–10 μM). Each biological replicate (*n* = 6 per group) represented the mean calculated from 3 individual DR5:GUS transgenic seedlings. The data are presented as mean ± standard deviation (SD). Different letters indicate statistically significant differences among the data as determined by one-way ANOVA (*p* < 0.05).

Pearson correlation analysis revealed a positive association between hypocotyl length and GUS activity regardless of IAA or TIBA treatment, indicating that auxin promotes hypocotyl length (Figure 3). Specifically, a strong positive correlation was exhibited between hypocotyl length and GUS activity (*r* = 0.980), indicating that longer hypocotyl length was consistently associated with higher GUS activity across photoperiod conditions (Figure 3a). At a low IAA concentration (0.001 μM), the correlation exhibited a strong positive correlation coefficient (*r* = 0.976), whereas higher concentrations of IAA (0.005 and 0.01 μM) reduced the correlation coefficient (*r* = 0.962 and 0.945, respectively), suggesting that auxin responsiveness reaches saturation under SD conditions with high auxin levels (Figure 3b–d). Similarly, TIBA treatment exhibited a progressive decrease in correlation with increasing concentration (1–10 μM), with correlation coefficients of *r* = 0.976, *r* = 0.933, and *r* = 0.832, respectively (Figure 3e–g). Moreover, exogenous IAA and TIBA treatments exhibited distinct photoperiod-dependent patterns (Figure 4). Under SD conditions, the hypocotyl length reached its maximum at 0.001 μM IAA concentration, consistent with the patterns of GUS activity. In contrast, the maximum response occurred at 0.005 μM IAA under MD conditions and at 0.01 μM IAA under LD conditions, both of which corresponded to the concentrations that produced the strongest GUS activity. These findings suggest that auxin sensitivity and the threshold for an optimal response are regulated by photoperiod conditions. Similarly, TIBA treatment induced a concentration-dependent increase in GUS activity, which was consistent with the inhibition of auxin efflux. In contrast to those under MD and SD conditions, hypocotyl length under LD conditions remained unaffected by TIBA treatment, indicating that auxin accumulation under LD condition was below the threshold required for transport inhibition to restrict growth. Taken together, these findings suggest that photoperiod concurrently regulates auxin accumulation and modulates the activation potential of auxin signaling pathways.

### Effect of photoperiod-dependent auxin accumulation on grafting success

**Figure 5.**
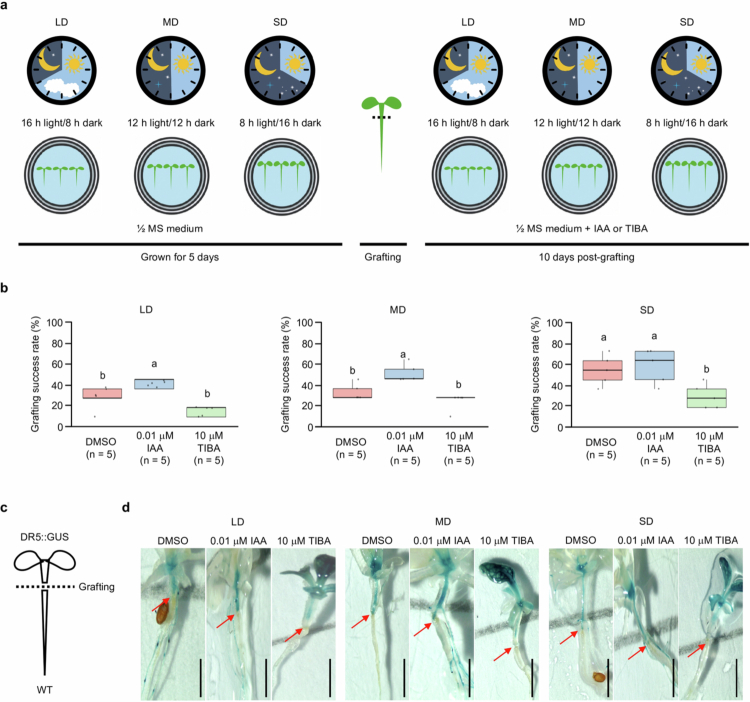
Effects of auxin and auxin transport inhibitor on the grafting success rate and auxin response at the graft junction under different photoperiod conditions in seedlings of *Arabidopsis thaliana*. (a) Schematic overview of the experimental design. Seedlings were grown vertically on ^1^/_2_ MS medium under different photoperiod conditions for 5 d. The seedlings were then grafted and transferred to ^1^/_2_ MS medium containing DMSO (mock control), 0.001 μM IAA, or 10 μM TIBA. The grafting success rate was evaluated 10 d postgrafting. (b) Quantification of the grafting success rate under each photoperiod and treatment condition. Each biological replicate (*n* = 5 per group) represented the mean grafting success rate calculated from 11 individual grafted seedlings that resulted in successful graft union formation (total of 55 grafts per condition). The data are presented as mean ±  standard deviation (SD). Different letters indicate statistically significant differences among the data as determined by one-way ANOVA (*p* < 0.05). (c) Schematic illustration of the DR5:GUS auxin-responsive reporter transgenic plants expressed in the scion used to monitor auxin signaling dynamics at the graft junction. (d) Representative histochemical GUS staining images of successfully grafted DR5:GUS transgenic scions. The red arrows indicate the graft union site. Scale bars = 1 mm.

To assess whether photoperiod-dependent auxin accumulation influences grafting efficiency, *A. thaliana* seedlings were initially grown for 5 d under different photoperiod conditions and subsequently transferred to ^1^/_2_ MS medium supplemented with DMSO, IAA, or TIBA while maintaining the same photoperiod conditions for an additional 10 d (Figure 5a). Under SD conditions, exogenous IAA treatment exhibited no significant change in grafting success, whereas TIBA treatment significantly reduced grafting success compared with that of the DMSO control. In contrast, under LD and MD conditions, IAA treatment significantly enhanced grafting success, whereas TIBA treatment exhibited no significant change in grafting success (Figure 5b). These findings suggest that endogenous auxin accumulation under SD conditions remains above the threshold required for grafting union formation, rendering additional auxin ineffective, whereas exogenous IAA treatment enhances grafting success under LD and MD conditions, implying that endogenous auxin accumulation under LD and MD conditions, is insufficient to reach the threshold for optimal grafting efficiency. To further investigate auxin transport and accumulation at the grafting junction, scions of DR5:GUS transgenic seedlings were grafted onto WT rootstocks under the same photoperiod conditions (Figure 5c). IAA treatment increased GUS activity at the graft junction across all photoperiod conditions, whereas TIBA treatment reduced GUS activity at the graft junction. These findings suggested that auxin transport and localized accumulation at the graft junction are critical for successful graft union formation (Figure 5d). Taken together, these findings suggest that photoperiod-dependent variation in auxin dynamics plays a critical role in regulating grafting success.

### Effect of postgrafting photoperiod alterations on grafting success

**Figure 6.**
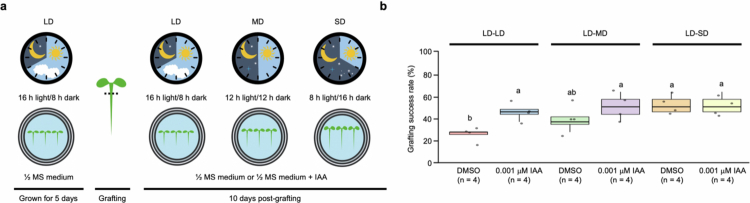
Effects of the postgrafting photoperiod and auxin treatment on the grafting success rate in seedlings of *Arabidopsis thaliana*. (a) Schematic overview of the experimental design. The seedlings were initially grown for 5 d under LD conditions before grafting. After grafting, the seedlings were transferred to ^1^/_2_ MS medium containing DMSO (mock control) or 0.001 μM IAA. Grafting success was evaluated 10 d postgrafting. (b) Quantification of the grafting success rate under different combinations of postgrafting photoperiods in the absence or presence of 0.001 μM IAA. LD–LD indicated that long-day conditions were maintained before and after grafting, LD–MD indicated a shift from long-day to medium-day conditions after grafting, and LD–SD indicated that a shift from long-day to short-day conditions after grafting. Each biological replicate (*n* = 4 per group) represented the mean grafting success rate calculated from 11 individual grafted seedlings that resulted in successful graft union formation (total of 44 grafts per condition). The data are presented as mean ±  standard deviation (SD). Different letters indicate statistically significant differences among the data as determined by one-way ANOVA (*p* < 0.05).

To determine whether photoperiod-regulated auxin dynamics influence grafting success, *A. thaliana* seedlings were grown under LD conditions for 5 d before grafting and subsequently exposed to shifted photoperiod conditions for 10 d with or without IAA treatment (Figure 6a). Postgrafting alterations in photoperiod conditions exhibited outcomes consistent with the effects of exogenous IAA treatment in enhancing grafting success (Figure 6b). Under continuous LD‒LD conditions, exogenous IAA treatment increased the grafting success rate. In contrast, transferring grafted seedlings from LD to either MD or SD conditions significantly increased grafting success regardless of exogenous IAA treatment. Notably, the grafting success rates observed under LD–MD and LD–SD conditions in the absence of IAA were comparable to those observed under continuous LD–LD conditions with exogenous IAA treatment. These findings suggest that photoperiod transitions act upstream of auxin biosynthetic and signaling pathways by modulating light-responsive transcriptional regulators.

## Discussion

Grafting is a fundamental strategy in modern agriculture to mitigate soil-borne diseases,^79^^,^^80^ increase crop productivity,^81^^,^^82^ and increase tolerance to abiotic stresses.^83^^,^^84^ Recent advances in plant grafting research have highlighted that the importance of graft union formation is governed by a hormone-regulated developmental process.^7^^,^^18^^,^^19^^,^^85^ Molecular and genetic analyses have also demonstrated that auxin, cytokinin, and gibberellin play a central role in a regulatory network that governs graft healing, callus formation, and vascular reconnection at the graft junction. Within this regulatory mechanism, our study provides novel evidence that photoperiod conditions act upstream of hormone signaling, which modulates auxin dynamics and grafting success. *A. thaliana* seedlings grown under SD conditions exhibited the highest grafting success rate, which was associated with hypocotyl elongation. In contrast, under LD and MD conditions, although *A. thaliana* seedlings exhibited significant differences in hypocotyl length before grafting, hypocotyl length was no longer significant after grafting, and the grafting success rate under both LD and MD conditions was lower than those under SD conditions (Figure 1). These findings suggest that SD condition sustains physiological signals that exceed the threshold required for hypocotyl elongation and graft union formation. In contrast, under the LD and MD conditions, such signals do not accumulate sufficiently after grafting, leading to the loss of hypocotyl elongation differences and limited graft union formation. Therefore, the photoperiod functions as a critical determinant of the grafting success rate by modulating the physiological state of *A. thaliana* seedlings in a manner conducive to tissue reprogramming and vascular reconnection.

Previous studies have demonstrated that auxin promotes graft healing by suppressing lateral bud outgrowth and increasing grafting success^86^ and promoting tissue adhesion, procambial cell division, and vascular reconnection during the graft healing process in *A. thaliana*.^21^^,^^87^ Furthermore, auxin signaling and biosynthesis are enhanced under SD conditions through the phytochrome–PIF4 module pathway.^88^^,^^89^ In this regard, a grafting experiment on *A. thaliana* seedlings with removed cotyledons, which are light-sensitive organs, revealed delayed vascular reconnection,^90^ suggesting that the photoperiod modulates the endogenous auxin levels required for graft healing. Histochemical GUS staining and DR5:GUS reporter analyses reinforced the mechanisms by exhibiting that photoperiod influences both auxin responsiveness and polar auxin transport (Figures 2–4). *A. thaliana* seedlings treated with exogenous IAA or TIBA revealed photoperiod-dependent differences in hypocotyl elongation and GUS activity. While exogenous IAA treatment increases auxin availability and can overcome the problem of suboptimal endogenous auxin levels, exogenous TIBA treatment blocks polar transport, limiting the ability of auxin signaling to induce elongation even at high overall auxin levels. Moreover, the strong correlation between DR5:GUS reporter GUS activity and hypocotyl elongation suggested that auxin signaling constitutes a direct determinant of the photoperiod-dependent growth response. However, at high concentrations of TIBA (5 and 10 μM), no significant difference in hypocotyl length was observed between LD and MD conditions, despite higher GUS activity under the MD condition than under the LD conditions. This discrepancy can be explained by the combined effects of photoperiod regulation and TIBA-induced inhibition of polar auxin transport. Under DMSO conditions as a control, photoperiod condition establishes differential auxin distribution patterns that promote hypocotyl elongation, whereas strong disruption of polar auxin transport negates the polar auxin distribution required for photoperiod-dependent growth. As a result, *A. thaliana* seedlings grown under MD conditions maintained higher GUS activity than those grown under LD conditions, suggesting that photoperiod signaling persists at the level of auxin synthesis or responsiveness. However, owing to the inability to effectively redistribute auxin, auxin signals were not transduced into hypocotyl elongation, a morphological response. Interestingly, the differential morphological response to exogenous IAA treatment under different photoperiod conditions was attributed to the extent of endogenous auxin accumulation. In our study, the term “threshold” refers to the minimal level of auxin accumulation and signaling required to promote efficient graft union formation. Under SD conditions, elevated endogenous auxin levels allow the threshold to be reached with small exogenous IAA treatment. In contrast, under LD and MD conditions, reduced endogenous auxin accumulation necessitates higher exogenous IAA treatment than under SD condition. These findings suggest that photoperiod regulates endogenous auxin dynamics, determining the efficiency of the response to exogenous IAA treatment. Furthermore, a previous study demonstrated that auxin enhances signaling by inhibiting the internalization step of PIN constitutive cycling, increasing the level of PIN protein at the plasma membrane, and promoting polar auxin transport through vesicle trafficking-dependent mechanisms.^75^ Consequently, these findings suggest that sufficient endogenous auxin accumulation and availability under SD conditions enable efficient polar auxin transport and signaling, establishing functional polar auxin transport that promotes hypocotyl elongation and enhances grafting success. In contrast, the LD and MD conditions lack sufficient endogenous auxin accumulation, limiting polar auxin transport and restricting these developmental processes.

Given this differential regulation of auxin dynamics under different photoperiod conditions, it was necessary to investigate whether exogenous IAA treatment or inhibition of polar auxin transport by TIBA could influence grafting success (Figure 5). Exogenous IAA treatment significantly enhanced grafting success under LD and MD conditions, whereas no significant difference was exhibited under SD conditions, suggesting that the auxin-dependent enhancement of grafting success is constrained by a threshold-type response, which is consistent with the saturation-type dose‒response of auxin signaling.^91^^,^^92^ In contrast, the inhibition of polar auxin transport by TIBA consistently reduced grafting success under different photoperiod conditions, suggesting an essential role of auxin transport in spatial redistribution and ensuring local accumulation at the graft junction. Histochemical GUS staining further confirmed that exogenous TIBA treatment suppressed GUS activity at the graft junction, establishing a direct link between auxin distribution and grafting success. These findings demonstrated that although auxin signaling and polar auxin transport constitute critical determinants of grafting success, the extent of auxin-mediated enhancement is modulated by photoperiod conditions, highlighting that light-regulated transcriptional networks define the capacity of auxin biosynthesis, distribution, and downstream signaling to promote graft union formation. It could be argued that the differential grafting success observed under different photoperiods could be attributed to variations in the daily light integral (DLI) or the technical ease of manipulating longer hypocotyls in SD-grown seedlings. However, our results demonstrated that exogenous IAA treatment significantly restored grafting success in LD-grown seedlings, even though these plants were exposed to a higher DLI and possessed shorter hypocotyls compared to SD plants. Crucially, since exogenous IAA was applied postgrafting, it did not alter the hypocotyl length at the time of the grafting procedure. This finding dissociates the physiological role of auxin from morphological traits, indicating that the photoperiodic enhancement of grafting success is driven primarily by the modulation of auxin dynamics rather than by cumulative photon energy or technical advantages in handling.

Building on this, the role of the photoperiod as an upstream regulator was further validated through a postgrafting photoperiod alteration experiment (Figure 6). *A. thaliana* seedlings transferred from the LD condition to the MD condition or the SD condition exhibited significantly higher grafting success regardless of exogenous IAA treatment, suggesting that photoperiod transitions recalibrate the light-responsive transcriptional regulators, resetting auxin biosynthesis and transport capacity. These findings reveal that photoperiod functions as a pivotal upstream regulator of auxin dynamics during graft healing. The contrasting activities of the light‒auxin regulatory cascade under LD and SD conditions provide a mechanistic explanation for the differential success of graft union formation. Under LD condition, activated PhyB increases HY5 accumulation by inhibiting the activity of the COP1/SPA1 E3 ubiquitin ligase, which degrades HY5,^93-95^ and increasing HY5 suppresses PIF4 activity, which activates YUC-mediated auxin biosynthesis,^41^^,^^88^^,^^96^^,^^97^ constraining auxin accumulation at the graft junction. In contrast, under SD condition, inactivated PhyB alleviates the inhibition of COP1/SPA1 E3 ubiquitin ligase activity to destabilize HY5, permitting PIF4 activation of YUC-mediated auxin biosynthesis and transport toward the graft junction. The localized auxin accumulation promotes cell division during callus formation through the induction of ANAC071 ^98^^,^^99^ and ERF115,^29^^,^^30^^,^^32^ and reinforces vascular reconnection by activating the PXY-WOX signaling network.^23^^,^^33^^,^^35^ The crosstalk between cytokinin and gibberellin promotes cell proliferation and vascular differentiation and enhances grafting success.^18^^,^^19^^,^^22^^,^^40^^,^^100^ Therefore, photoperiod regulation functions as a critical determinant of grafting success by orchestrating light-dependent transcriptional networks that govern auxin dynamics and downstream developmental programs essential for tissue adhesion, callus proliferation, and vascular reconnection.

This photoperiod-dependent modulation of auxin availability appears to be a decisive factor that determines whether graft-induced cell division, proliferation, and vascular reconnection proceed efficiently. Importantly, our results suggest that auxin functions as part of an integrated hormonal network rather than acting independently. Elevated auxin levels activate the transcription factors ANAC071 and ERF115, which is consistent with previous studies indicating their roles as central regulators of regenerative cell division during tissue adhesion and callus formation.^29^^,^^30^^,^^32^^,^^98^^,^^99^ Simultaneous activation of PXY-WOX signaling provides mechanistic evidence that auxin is a principal regulator of cambial proliferation and vascular differentiation during graft union formation.^23^^,^^33^^,^^35^ Furthermore, the potentiation of these auxin-regulated modules by cytokinin and gibberellin indicates that graft healing is not mediated by a linear auxin response but instead emerges from a hierarchically organized network of hormonal crosstalk, in which multiple phytohormones act synergistically to coordinate cell differentiation and vascular formation.^18^^,^^19^^,^^22^^,^^40^^,^^100^

## Conclusion

This study demonstrates that the photoperiod functions as a pivotal upstream regulator of graft healing by modulating auxin biosynthesis, distribution, and responsiveness. Under SD conditions, endogenous auxin levels are maintained above the threshold required for hypocotyl elongation and grafting success, whereas under LD and MD conditions, they failed to maintain sufficient auxin dynamics to support efficient graft union formation. Moreover, postgrafting photoperiod alteration restored the grafting success rate, underscoring the dynamic plasticity of light-responsive transcriptional regulators in resetting auxin-mediated regeneration. Therefore, these findings provide a conceptual framework in which photoperiod is established as an environmental determinant of regenerative competence. By linking environmental cues to the hormonal network, our study advances the understanding of the diverse outcomes of grafting and suggests translational opportunities for enhancing grafting success in crops through targeted modulation of photoperiod conditions and hormone signaling pathways.

## Supplementary Material

Supplementary material Supplementary material

## Data Availability

The data that support the findings of this study are available from the corresponding author upon reasonable request.
