# Supplementary material for: Short-day photoperiod enhances graft success by increasing auxin accumulation
Source: Plant Signal Behav. 2026 Mar 31;21(1):2648973. doi: 10.1080/15592324.2026.2648973 (PMC13048592; doi:10.1080/15592324.2026.2648973)
Supplement: Supplementary material [file KPSB_A_2648973_SM0813.docx]

**
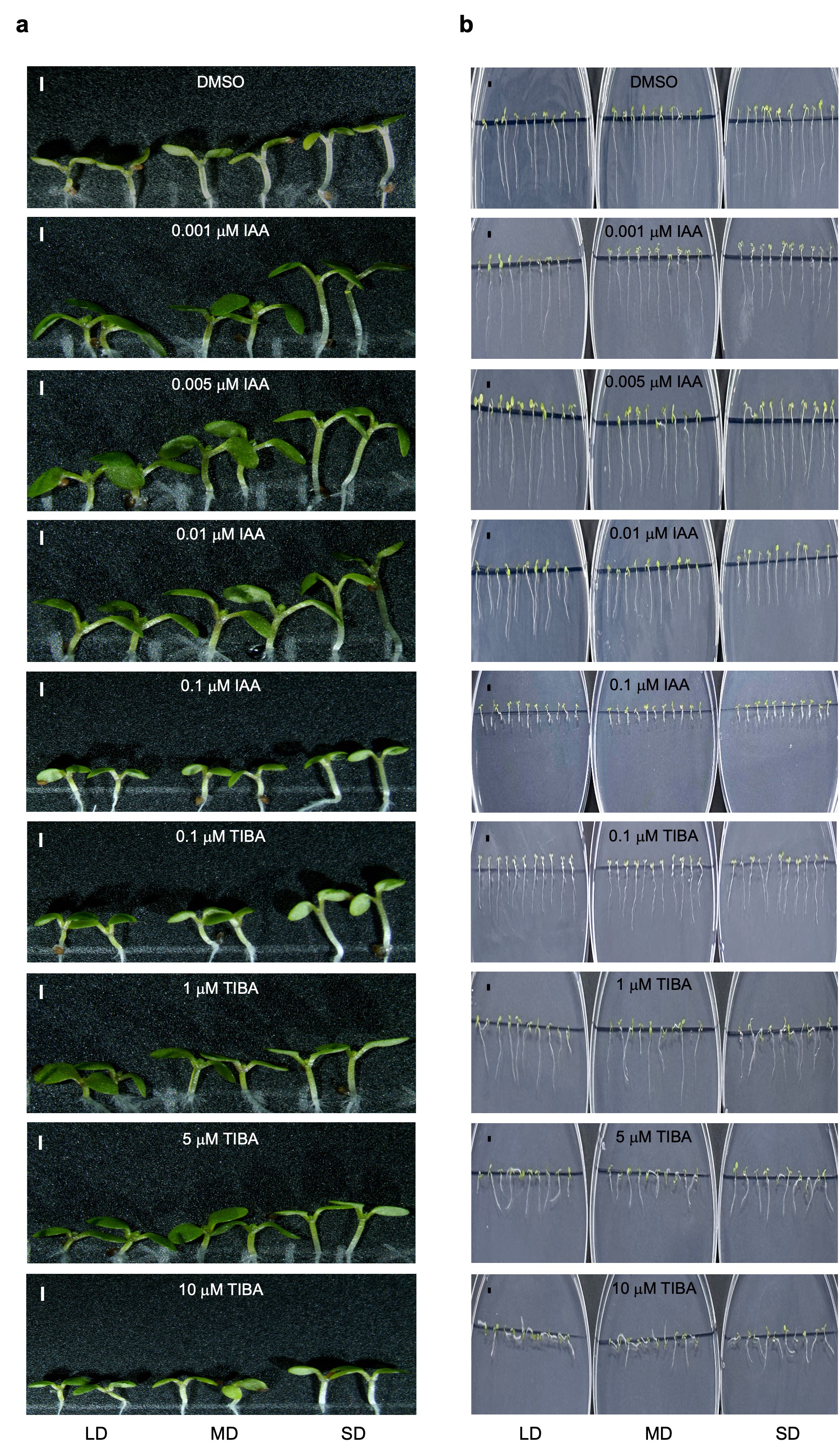
**

**Supplemental Figure 1** Determination of optimal IAA and TIBA concentrations for photoperiod-dependent hypocotyl length in seedlings of *Arabidopsis thaliana*. (a) Representative images of 5-day-old seedlings grown under different photoperiod conditions on ^1^/_2_ MS medium containing a range of IAA (0.001-0.1 μM) or TIBA (0.1-10 μM) concentrations. (b) Representative images of vertically grown 5-day-old seedlings treated with a range of IAA (0.001-0.1 μM) or TIBA (0.1-10 μM) concentrations under different photoperiod conditions. These images were acquired to visualize the overall seedlings’ morphology under each treatment condition. Scale bars = 1 mm.

**Supplemental Table 1** List of primers used in this study

| Primer name | Forward (5’ **→** 3’) | Amplicon size (bp) | Purpose |
| --- | --- | --- | --- |
| 35S_minimal_F | CTATTGCAGCAATTTAAATCATTTC | 226 bp | Verification of presence of  the DR5::GUS expression cassette |
| GUSstart_R | CCCACCAACGCTGATCAATTC |  |  |
